# 251. Poor Outcomes in the Treatment of Coagulase-Negative Staphylococci Periprosthetic Joint Infections

**DOI:** 10.1093/ofid/ofab466.453

**Published:** 2021-12-04

**Authors:** Ayden Case, Lefko T Charalambous, Jessica Seidelman, Edward Hendershot, William Jiranek, Michael Bolognesi, Thorsten Seyler

**Affiliations:** 1 Duke University, Durham, North Carolina; 2 Duke University Medical Center, Dept. of Orthopaedics, Durham, North Carolina

## Abstract

**Background:**

Coagulase-negative staphylococci (CoNS) are a common skin flora often considered lab contaminants, but these pathogens can also be the cause of periprosthetic joint infections (PJIs). The role of these organisms in PJIs is not well characterized, with little data relating to treatment outcomes. We sought to evaluate success at one year for patients undergoing treatment for a CoNS PJI.

**Methods:**

This is a retrospective cohort study of adults at a tertiary academic center from 2009 to 2020 with CoNS PJI. An institutional database was queried to identify potential patients and manually reviewed by two infectious disease specialists to confirm inclusion. Variables included sex, follow-up time, procedure type, age, race, Elixhauser score, success at one year, failure organism, and revisions. Both univariate and descriptive statistics were used to assess findings.

**Results:**

We identified 61 patients with a CoNS PJI. The cohort was 50.8% male, with 49 patients identifying as Caucasian (80.3%), and 10 as African American (16.4%). The median age was 65.0 years old, the median Elixhauser score was 3.0, and the average follow-up time was 24.4 months. Of the 61 patients in the cohort, 24 underwent successful treatment (39.3%) at one year, and 37 failed treatment (60.7%). Within the failure group, 19 experienced persistence of the same organism (51.4%), 11 were infected by another organism (29.7%), and 28 underwent a revision surgery secondary to failure (76.9%). When stratified by treatment procedure after initial PJI, 26 (41.7%) received debridement, antibiotics, and implant retention (DAIR) whereas 35 (58.3%) underwent resection. Treatment success was not significantly different between the two procedures (p=0.964).

Summary of Treatment Success for CoNS PJI

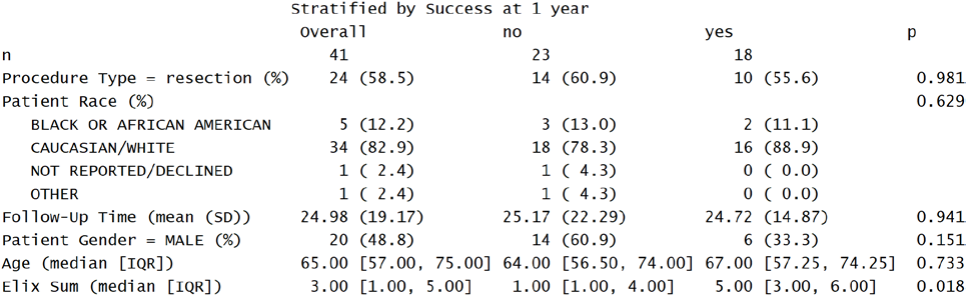

**Conclusion:**

These results indicate that the success rate of treatment for CoNS PJI is less than for other organisms, such as coagulase-positive staphylocci. These results provide a focus for future research and clinical management of PJIs resulting from CoNS.

**Disclosures:**

**William Jiranek, MD**, **Depuy Synthes** (Other Financial or Material Support, Royalty/Licensing) **Michael Bolognesi, MD**, **Heron Therapeutics, Inc.** (Consultant)**Total Joint Orthopedics, Inc.** (Other Financial or Material Support, Royalty/Licensing)**Zimmer Biomet Holdings, Inc.** (Other Financial or Material Support, Royalty/Licensing) **Thorsten Seyler, MD/PhD**, **Depuy Synthes** (Other Financial or Material Support, Resident Educational Support)**Extrel Therapeutics** (Board Member, Shareholder)**Heraeus Medical** (Consultant)**MiCare Path** (Board Member, Shareholder)**OREF** (Grant/Research Support)**Pattern health** (Board Member)**Restor3D** (Other Financial or Material Support, Royalties)**Smith+Nephew, Inc.** (Grant/Research Support, Speaker’s Bureau)**Stryker** (Other Financial or Material Support, Resident Educational Support)**Total Joint Orthopedics, Inc.** (Consultant)**Wolters Kluwer Health** (Other Financial or Material Support, Royalties)**Zimmer Biomet** (Grant/Research Support)

